# Genome-wide long non-coding RNA screening, identification and characterization in a model microorganism *Chlamydomonas reinhardtii*

**DOI:** 10.1038/srep34109

**Published:** 2016-09-23

**Authors:** Hui Li, Yuting Wang, Meirong Chen, Peng Xiao, Changxing Hu, Zhiyong Zeng, Chaogang Wang, Jiangxin Wang, Zhangli Hu

**Affiliations:** 1 Guangdong Technology Research Center for Marine Algal Bioengineering, Guangdong Key Laboratory of Plant Epigenetic, College of Life Sciences, Shenzhen University, Shenzhen 518060, P. R. China; 2 Shenzhen Key Laboratory of Marine Bioresource & Eco-environmental Science, College of Life Sciences, Shenzhen University, Shenzhen 518060, P. R. China; 3 Key Laboratory of Optoelectronic Devices and Systems of Ministry of Education and Guangdong Province, College of Optoelectronic Engineering, Shenzhen University, Shenzhen 518060, P. R. China

## Abstract

Microalgae are regarded as the most promising biofuel candidates and extensive metabolic engineering were conducted but very few improvements were achieved. Long non-coding RNA (lncRNA) investigation and manipulation may provide new insights for this issue. LncRNAs refer to transcripts that are longer than 200 nucleotides, do not encode proteins but play important roles in eukaryotic gene regulation. However, no information of potential lncRNAs has been reported in eukaryotic alga. Recently, we performed RNA sequencing in *Chlamydomonas reinhardtii*, and obtained totally 3,574 putative lncRNAs. 1440 were considered as high-confidence lncRNAs, including 936 large intergenic, 310 intronic and 194 anti-sense lncRNAs. The average transcript length, ORF length and numbers of exons for lncRNAs are much less than for genes in this green alga. In contrast with human lncRNAs of which more than 98% are spliced, the percentage in *C. reinhardtii* is only 48.1%. In addition, we identified 367 lncRNAs responsive to sulfur deprivation, including 36 photosynthesis-related lncRNAs. This is the first time that lncRNAs were explored in the unicellular model organism *C. reinhardtii*. The lncRNA data could also provide new insights into *C. reinhardtii* hydrogen production under sulfur deprivation.

A considerable portion of the genome of eukaryotes can be transcribed to RNAs, but will not be translated to proteins. These non-coding RNAs (ncRNAs) consist of housekeeping, regulatory and functional unknown ncRNAs. Regulatory ncRNAs are usually classified as small non-coding RNAs and long non-coding RNAs (lncRNAs) according to their lengths^1^,^2^. Little was known about the function of lncRNA for a long time, but the discovery of the function of HOTAIR lifts ncRNAs to new levels^3^. HOTAIR is a 2.2 kb non-coding RNA, which represses transcription of the *HOXD* locus *in trans* across 40 kb^4^. In fact, previous studies have showed that *HOX* gene clusters play important roles in embryonic development. After the discovery of HOTAIR, lncRNAs began come into the spotlight. Now it is well known that lncRNAs play important roles in cell differentiation and development^1^,^5^,^6^, silencing gene expression in X-chromosome^7^, neurological diseases occurrence^8^, cancer progression^9^,^10^ and immune response genes mediation^11^,^12^.

In plants, lncRNAs express differentially in various organs and under different treatment conditions, which indicates lncRNAs can modulate gene activity during development and in response to external stimuli^13^. For instance, in *Arabidopsis* 6,480 intergenic transcripts were classified as long intergenic non-coding RNAs (lincRNAs). A subset of these lincRNAs express organ-specifically, whereas others are responsive to biotic and/or abiotic stresses^14^. Interestingly, a large number of *Arabidopsis* long non-coding natural antisense transcripts responded to light are dynamically correlated with histone acetylation^15^. Very recently, genome-wide screening and functional analysis in rice identified a set of lncRNAs that are involved in the sexual reproduction^16^. A 1.2 kb rice lncRNA, referred as long-day-specific male-fertility-associated RNA (LDMAR) regulates photoperiod-sensitive male sterility (PSMS), which is the essential component of hybrid rice^17^. In addition, there are 1,704 high-confident lncRNAs identified in maize, and the tissue-specific expression of maize lncRNAs is more significant than that of filtered genes^18^.

As to microorganisms, lncRNAs were proved to mediate sporulation via chromatin regulation in both budding yeast *Saccharomyces cerevisiae* and fission yeast *Schizosaccharomyces pombe*^19^,^20^. Subset of ncRNAs, natural antisense transcripts (NATs) had been genome-wide investigated in the ascomycetes *S. cerevisiae*, *Candida albicans*, *Aspergillus flavus*, *Magnaporthe oryzae*, *Tuber melanosporum* and *S. pombe*, as well as the basidiomycetes *Cryptococcus neoformans*, *Ustilago maydis* and *Schizophyllum commune*. The results demonstrated that a large number of NATs existed in various fungi^21^. In the model filamentous fungus *Neurospora crassa*, 939 lncRNAs have been successfully identified based on the results of RNA sequencing. Interestingly, these lncRNAs could be regulated by different environmental stimuli^22^.

Microalgae are regarded as the most promising biofuel candidates and extensive metabolic engineering are being conducted to reduce the biofuel production cost but very few improvements are achieved yet. Based on the regulatory functions found in other higher plants, lncRNA investigation and manipulation may provide new insights and solutions for this issue. The *Chlamydomonas reinhardtii* is a unicellular green alga, which is a model organism in the study of chloroplast-based photosynthesis, the structure and function of eukaryotic flagella, as well as many metabolic processes^23^. *C. reinhardtii* is also an ideal model organism to study hydrogen metabolism in photosynthetic eukaryotes^24^. Sulfur deprivation has been proved to be highly correlated to hydrogen photoproduction, and it leads to sustained hydrogen production in *C. reinhardtii*^24^. Transcriptome and proteome analyses indicated that sulfur deprivation affects massive pathways including sulfur metabolism, cell wall structure, photosystems, protein biosynthetic apparatus, molecular chaperones and 20 S proteasomal components^25^,^26^,^27^. It was previously demonstrated that hydrogen production can be regulated by an artificial non-coding RNA miRNA (amiRNA) targeting OEE2 encoded gene (a photosystem II related protein, oxygen evolving enhancer)^28^. This suggested a prospective way for continuous hydrogen production in green algae using regulatory ncRNAs. LncRNAs might be one of the potential solutions for above issues, which explain why we first used the sulfur-deprived *C. reinhardtii* cells when screening the whole genome for lncRNAs.

The complete genome of *C. reinhardtii* had been sequenced in ref. 23, and miRNAs had also been found in *C. reinhardtii*^29^,^30^,^31^. However, the lncRNAs still remains completely unknown in any microalga. In this study, we have carried out a genome-wide scanning using cutting edge high-throughput RNA-seq to discover and characterize the lncRNAs in *C. reinhardtii*. LncRNA target genes were also predicted to annotate lncRNA functions. Finally, the lncRNAs expression changes in sulfur-replete and sulfur-deprived conditions were explored as well.

## Results

### Genome-wide identification of lncRNAs in *C*. *reinhardtii*

**Figure 1:**
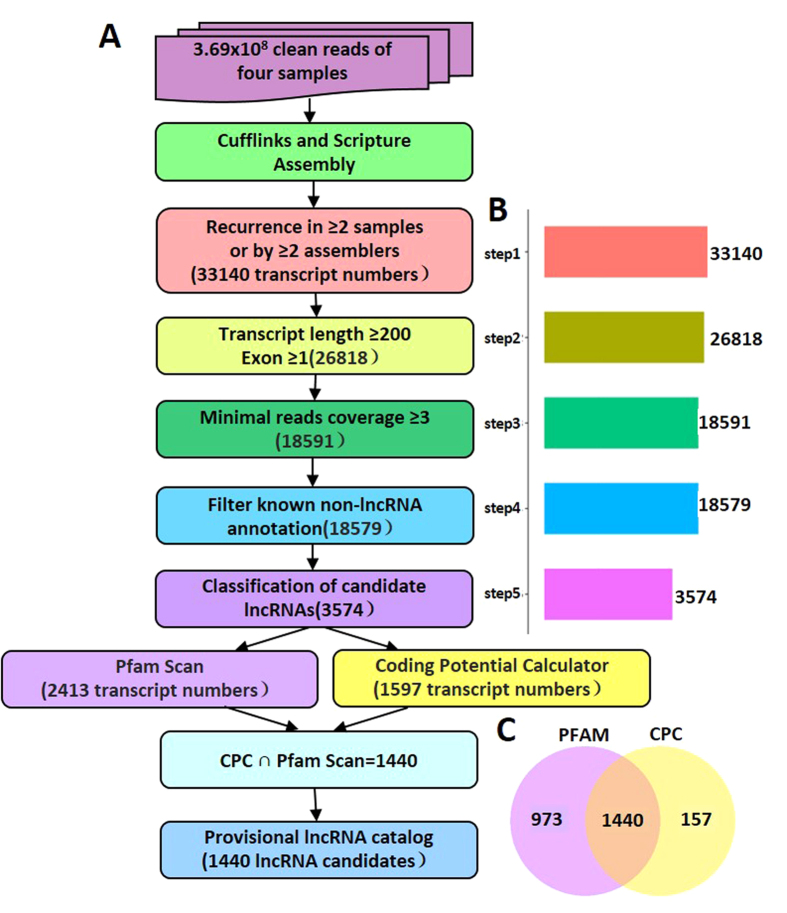
An integrative computational pipeline for the systematic identification of lncRNAs in *C. reinhardtii*. (**A**) Informatics pipeline for the identification of lncRNAs in *C. reinhardtii*. (**B**) The candidate transcripts numbers of the five filtering steps. (**C**) Venn chart showing the numbers of candidate lncRNAs filtered by the PFAM, CPC assemblies or by both assemblies. PFAM: Pfam Scan, CPC: Coding Potential Calculator.

To identify lncRNAs in *C*. *reinhardtii*, we performed RNA-seq using *C. reinhardtii* cells cultured under sulfur-replete and sulfur-deprived conditions. We performed four samples in total, including two sulfur-replete samples and two sulfur-deprived samples. Cells were cultured in TAP + S (with sulfate 40.55 mg/L) and TAP-S (the S-salts was replaced by their chloride counterparts) medium respectively. Total RNA was isolated using RNAiso and cDNA libraries were constructed for sequencing with NEBNext^®^ Ultra™ Directional RNA Library Prep Kit for Illumina^®^ (NEB, USA) according to the manufacturer’s instructions, respectively. 194,241,412 and 174,811,628 clean reads were obtained from sulfur-replete (+S) and sulfur-deprived (−S) libraries, respectively (Fig. 1A). The sequences were mapped to *C. reinhardtii* genome retrieved from NCBI (ftp://ftp.ensemblgenomes.org/pub/plants/release-23/fasta/chlamydomonas_reinhardtii/dna/). Details of sequencing and mapping steps can be found in Supplementary Data S1 and Table S1. Identification of lncRNAs was executed according to the pipeline shown in Fig. 1. Briefly, the data were firstly filtered using five basic principles: (1) Recurrence in ≥ 3 samples or by ≥ 2 assemblers; (2) Transcript length ≥ 200, and exon number ≥ 1; (3) Minimal reads coverage ≥ 3; (4) Filter known non-lncRNA annotation; (5) Classification of candidate lncRNAs. As a result, 3,574 sequences were obtained after the sifting (Fig. 1B). To effectively distinguish protein-coding and non-coding sequences, coding potential filtering was performed subsequently according to CPC (Coding Potential Calculator) and Pfam Scan (v1.3). By this way, 2,413 and 1597 candidate lncRNAs were predicted by CPC and Pfam Scan, respectively. Finally 1,440 lncRNAs were obtained in the intersection of CPC and Pfam Scan (Fig. 1C). The sequences of all 1,440 lncRNAs identified by CPC and Pfam were listed in Supplementary Data S2.

### Validation of transcription levels of *C. reinhardtii* lncRNAs

**Figure 2:**
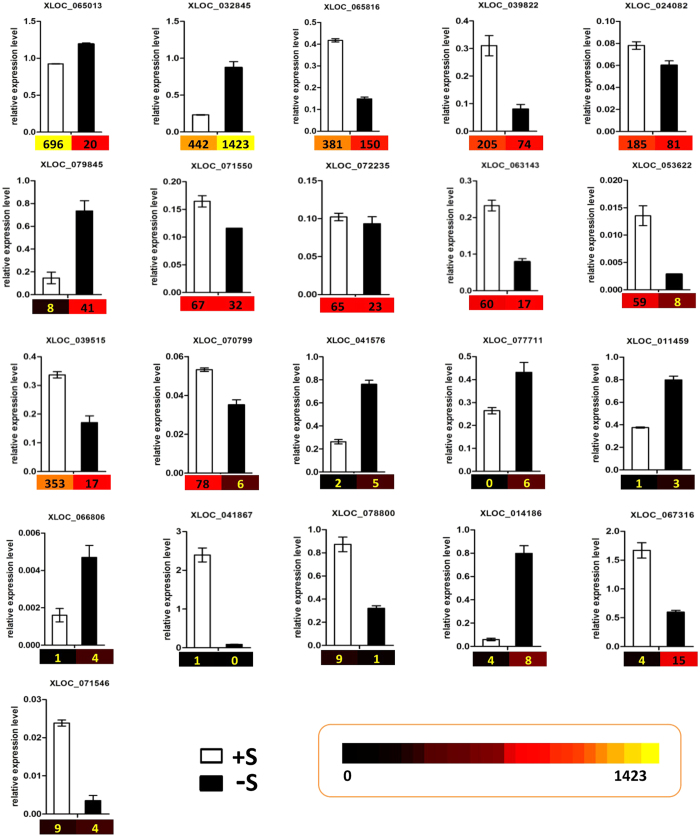
qRT-PCR validation of putative lncRNAs in *C. reinhardtii*. 21 putative lncRNAs, including 17 lincRNAs and 4 intronic lncRNAs are selected for quantitative PCR validation. *U4* was used as the reference gene. Colors and numbers under the bar charts refers to the FPKM of genes.

To confirm the expression of *C. reinhardtii* lncRNAs and their response to sulfur-deprived stress, quantitative Real-Time PCR (qRT-PCR) analysis was applied to verify the results of the high-throughput RNA-seq sequencing. Total RNA extracted from the same samples as RNA-seq used for *C. reinhardtii* cells cultured under sulfur-replete and sulfur-deprived conditions was converted to cDNA by reverse transcription. Real-Time PCR was next employed to validate the expression levels of 21 lncRNAs selected from the RNA-seq results at random (Fig. 2). The U4 was used as the internal control for quantification^31^,^32^.

LncRNAs are classified into four types according to their genomic location and context^33^,^34^. In this study we detected three main types of lncRNAs in *C. reinhardtii*: intergenic lncRNAs (lincRNAs), intronic lncRNAs and anti-sense lncRNAs.

Totally 21 putative lncRNAs, including 17 lincRNAs and 4 intronic lncRNAs were randomly selected for quantitative PCR validation. The results demonstrated that in most cases results of qRT-PCR were consistent with those of RNA-seq. This correlation confirmed that the results of RNA-seq technique are reliable. The expression levels of 18 lncRNAs matched these of high throughput sequencing data. However, 3 of the chosen low read lncRNAs did not match the RNA-seq results. We deduce that it is likely due to the low abundance of lncRNAs and the amplification efficiency.

### Characterization of *C. reinhardtii* lncRNAs

**Figure 3:**
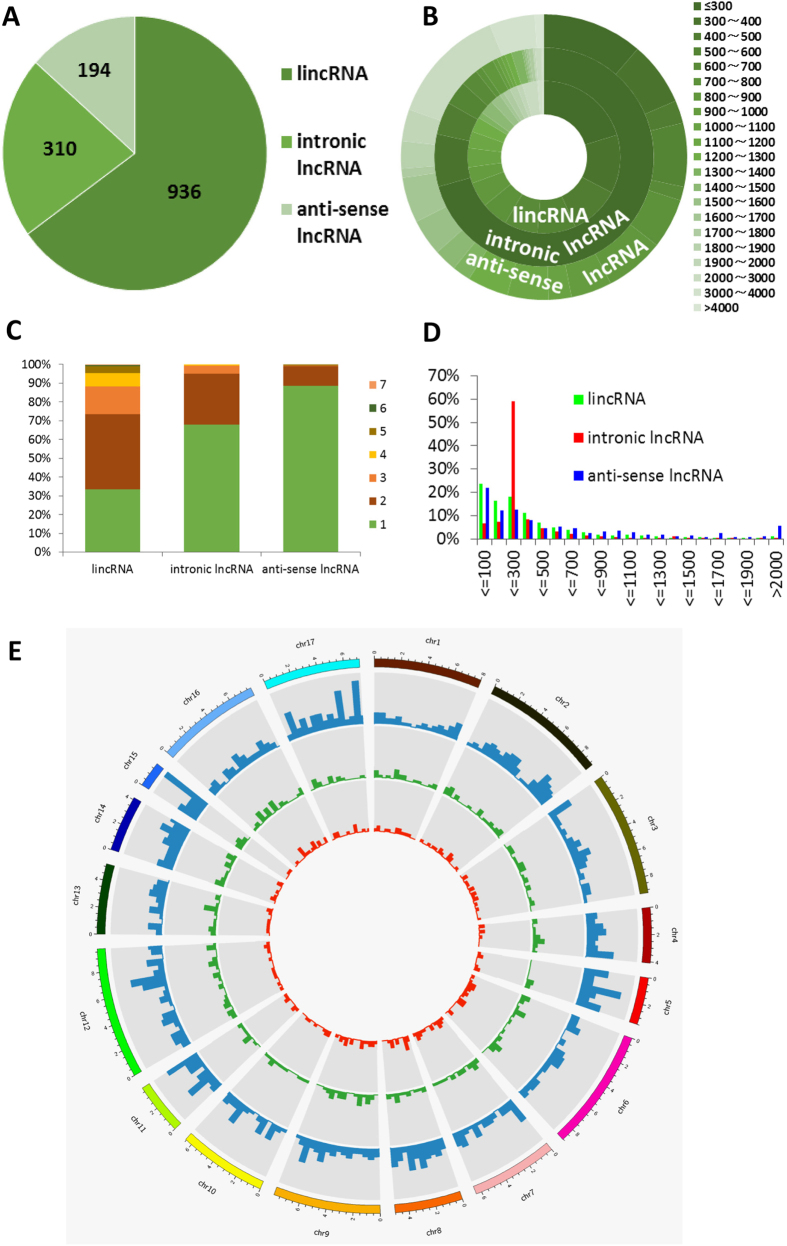
Characteristics of *C. reinhardtii* lncRNAs. (**A**) Numbers of lincRNAs, intronic lncRNAs and anti-sense lncRNAs in *C. reinhardtii*. (**B**) Transcript length distribution of lincRNAs, intronic lncRNAs and anti-sense lncRNAs. (**C**) The number of exons per transcript for lncRNAs. (**D**) Exon length distribution of lincRNAs, intronic lncRNAs and anti-sense lncRNAs. (**E**) Distribution of lncRNAs along each chromosome. The numbers of lincRNAs (outer circle, depicted in blue), intronic lncRNAs (middle circle, depicted in green) and anti-sense lncRNAs (inner circle, depicted in red) in physical bins of 500 kb for each chromosome.

For the first time, characteristics and transcription patterns of *C. reinhardtii* lncRNAs were investigated in this study. The 1440 newly identified *C. reinhardtii* lncRNAs included 936 lincRNAs, 310 intronic lncRNAs and 194 anti-sense lncRNAs (Fig. 3A). LincRNAs comprise the major part of total lncRNAs (65% of the total *C. reinhardtii* lncRNAs). Full-length *C. reinhardtii* lncRNA transcripts (median length of 509 nucleotides) are longer than *Arabidopsis* lncRNA transcripts (median length of 285 nucleotides), but shorter than human (median length of 592 nucleotides) and rice (median length of 852 nucleotides)^19^,^20^,^21^,^35^. Interestingly, anti-sense lncRNAs with about 1200 nucleotides in length were found to be the longest transcripts among the three types of lncRNAs in *C. reinhardtii*. In contrast, a majority of intronic lncRNAs are shorter than 300 nucleotides (Fig. 3B). When we compared the exon number in different type of lncRNA of *C. reinhardtii*, 67.9% of intronic lncRNAs and 88.6% of anti-sense lncRNAs have only one exon, while lincRNAs usually have one or two exons (33.3% of lincRNAs have one exon and 40.3% of lincRNAs have two exons) (Fig. 3C). In addition, more than 20% of *C. reinhardtii* lincRNAs and anti-sense lncRNAs exons are shorter than 100 nucleotides, but almost 60% of intronic lncRNAs exons distribute among the regions of 200–300 nucleotides (Fig. 3D). LncRNAs evenly distributed in each chromosome and lincRNAs do not show significant chromosome location preference either (Fig. 3E).

In contrast with more than 98% human lncRNAs are spliced^34^, only 48.1% of spliced *C. reinhardtii* lncRNAs were observed in our study. Interestingly, the percentage of spliced *C. reinhardtii* lincRNAs (66.7%) is higher than that of rice spliced lincRNAs (46.5%)^21^.

**Figure 4:**
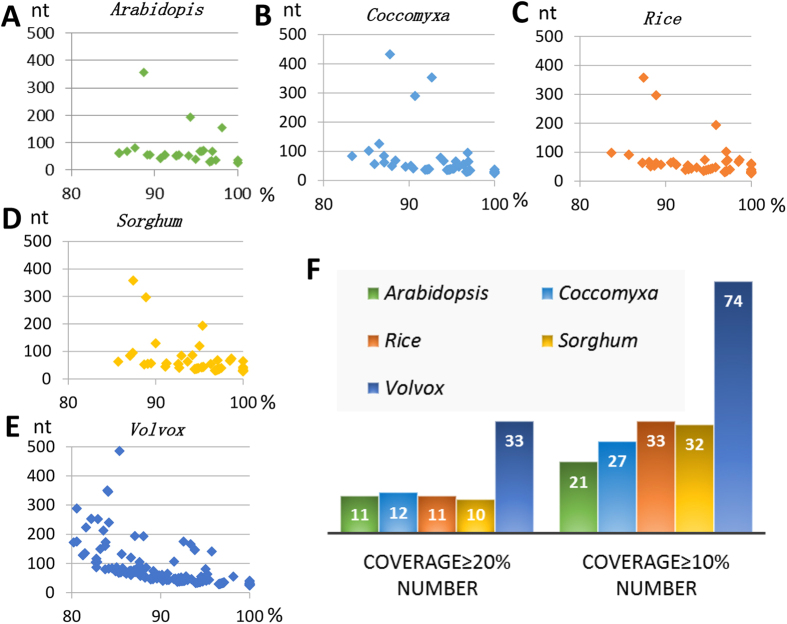
The *C. reinhardtii* lncRNAs that are conserved in Arabidopsis thaliana, Homo sapiens, Coccomyxa subellipsoidea, Oryza sativa, Sorghum bicolor and Volvox carteri. All the *C. reinhardtii* lncRNAs are blasted with the genomes of Arabidopsis thaliana (**A**), Coccomyxa subellipsoidea (**B**), Oryza sativa (**C**), Sorghum bicolor (**D**) and Volvox carteri (**E**). X axis: percentage of identity. Y axis: align length. E value < 1.0E-5. (**F**) Conserved lncRNAs numbers with more than 20% or 10% coverage regions.

The conservation of lncRNAs is considered lower than that of protein coding genes in comparisons between species. All *C. reinhardtii* lncRNAs were blasted against the genomes of *Arabidopsis thaliana*, *Coccomyxa subellipsoidea*, *Oryza sativa*, *Sorghum bicolor* and *Volvox carteri* (Fig. 4). In *C. reinhardtii* only 38 lncRNAs were predicted to be conserved with that of *Arabidopsis*, while 169 lncRNAs shared homology with *Volvox carteri* genome (Table 1). The entire list of all conserved lncRNAs can be found as Supplementary Data S3. The *C. reinhardtii* have longer conserved sequences compared with *Coccomyxa subellipsoidea* and *Volvox carteri* (Table 1), which indicated that *C. reinhardtii* may have higher conservation with these two species in terms of lncRNA conservation. Besides, the coverage value referred to percentage of conserved sequence regions in full length lncRNAs was also investigated to predict the most homologue specie with *C. reinhardtii*. LncRNAs with more than 10% or 20% coverage were summarized, and the results showed that *C. reinhardtii* possessed most conserved lncRNAs sequences when compared with *Volvox carteri* at both over 10% and over 20% coverage levels. This result suggested that *C. reinhardtii* lncRNAs were most conserved with *V. carteri*.

### Basic property comparison of lncRNAs and mRNAs

**Figure 5:**
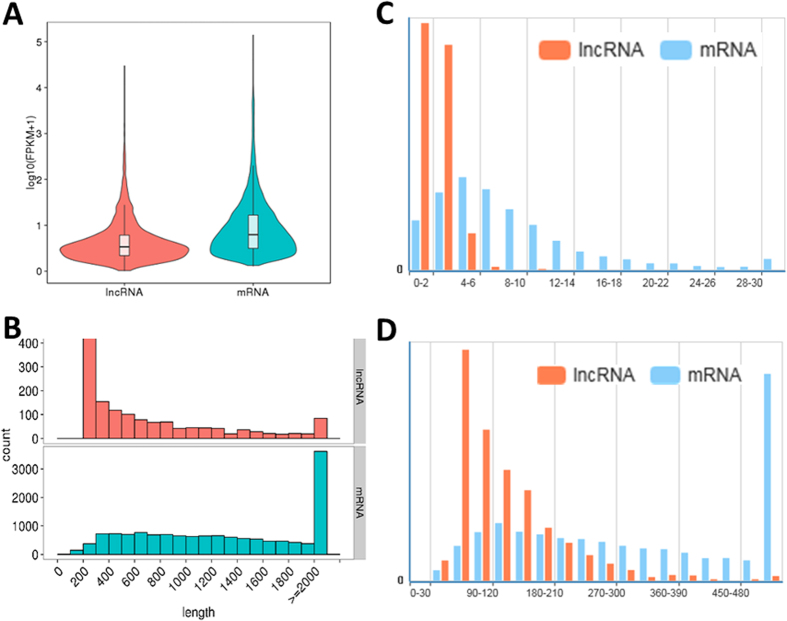
Comparison of *C. reinhardtii* lncRNAs and mRNAs. (**A**) Expression levels of lncRNAs and mRNAs. (**B**) Transcript length of lncRNAs and mRNAs. (**C**) Comparison of exon numbers of lncRNAs and mRNAs. Y axis: frequency count. (**D**) Comparison of ORFs length of lncRNAs and mRNAs. Y axis: frequency count.

The properties such as transcript abundance, lengths, exon numbers and ORFs (Open Reading Frames) of *C. reinhardtii* lncRNAs and mRNAs have also been compared under the same conditions (Fig. 5). The data of FPKM (expected number of fragments per kilobase of transcript sequence per million mapped reads) represented the abundance of lncRNAs were lower than those of mRNAs in RNA-seq samples, indicting lncRNAs were less transcribed (Fig. 5A). Also, we found that the lengths of lncRNAs were usually shorter than mRNAs. For instance, the lengths of most *C. reinhardtii* lncRNAs were from 200 to 300 nucleotides, while most mRNAs were longer than 2,000 nucleotides (Fig. 5B). Moreover, fewer exons existed in lncRNAs than in mRNAs. For example, most lncRNAs have fewer than six exons, while mRNAs have more exons and exon numbers distribute in a wider range instead. Some mRNAs have as many as thirty exons (Fig. 5C). The *C. reinhardtii* lncRNAs also have shorter (60–90 nucleotides) ORFs than those of mRNAs, while most mRNAs ORFs are more than 500 nucleotides (Fig. 5D).

### Alternative splicing events

As one of the most reported functional bioprocesses by lncRNAs, alternative splicing events^36^,^37^ based on the RNA-seq data were investigated in *C. reinhardtii*. The numbers of different alternative splicing events were calculated and recognized as 12 types: (1) TSS: Alternative 5′ first exon; (2) TTS: Alternative 3′ last exon; (3) SKIP: Skipped exon; (4) XSKIP: Approximate SKIP; (5) MSKIP: Multi-exon SKIP; (6) XMSKIP: Approximate MSKIP; (7) IR: Intron retention; (8) XIR: Approximate IR; (9) MIR: Multi-IR; (10) XMIR: Approximate MIR; (11) AE: Alternative exon ends (5′, 3′, or both) and (12) XAE: Approximate AE. No difference in numbers of all kinds of alternative splicing events could be identified between the sulfur-replete and sulfur-deprived *C. reinhardtii* samples (see Supplementary Figure S1). As a consequence, further study is needed for the alternative splicing events case by case in *C. reinhardtii*.

### LncRNAs responsive to sulfur deprivation

**Figure 6:**
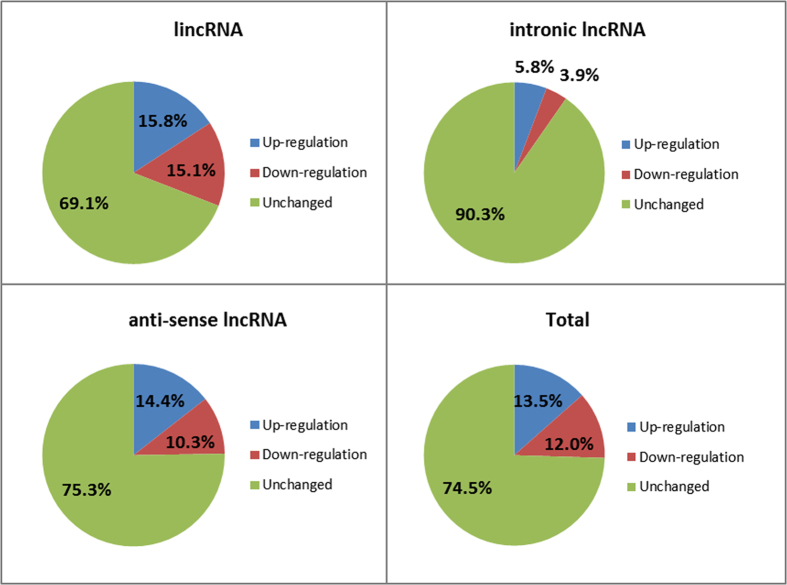
Percentage of changed *C. reinhardtii* lncRNAs under sulfur-deprived condition.

**Figure 7:**
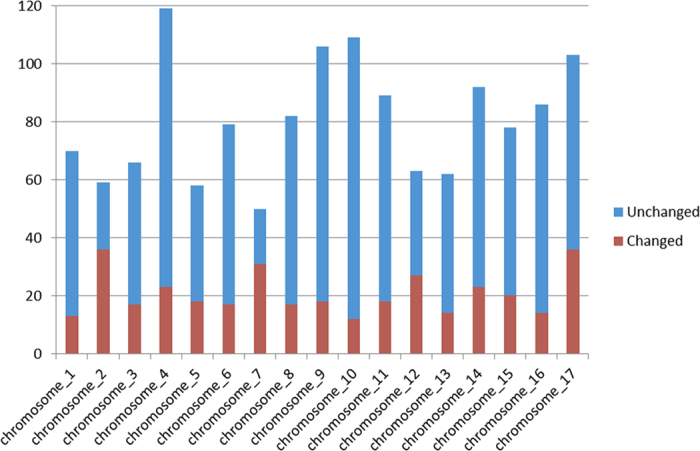
Chromosome distribution of changed and unchanged *C. reinhardtii* lncRNAs under sulfur-deprived condition. The Y axis represents numbers of lncRNAs.

Totally 367 lncRNAs responsive to sulfur deprivation (see Supplementary Data S4), including 194 up-regulated lncRNAs and 173 down-regulated lncRNAs were identified in this study (Table 2), which were classified to 289 lincRNAs (78.7%), 30 intronic lncRNAs (8.2%) and 48 anti-sense lncRNAs (13.1%). The lncRNAs with up-regulated levels after sulfur deprivation were more than down-regulated ones in all three types of lncRNAs. The proportion of differentially expressed lncRNAs under sulfur-deprived conditions was also analyzed. For instance, 30.9% of lincRNAs changed after sulfur deprivation, and the numbers of up-regulated and down-regulated lincRNAs were very similar. However, only 9.7% intronic lncRNAs were responsive to sulfur deprivation (Fig. 6). The results showed that the lincRNAs were more responsive to sulfur deprivation, while intronic lncRNAs were less affected. Interestingly, when we looked at the chromosome preference of lncRNAs under sulfur deprivation, more than 60% lncRNAs on chromosome 2 and chromosome 7 were responsive to sulfur deprivation, respectively. In contrast, only 11.0% lncRNAs on chromosome 10 were changed under sulfur-deprived condition (Fig. 7).

Among the 367 lncRNAs responsive to sulfur deprivation, 6 lncRNAs and 10 lncRNAs were only expressed under sulfur-replete (Table 3) and sulfur-deprived (Table 4) condition, respectively. The top 10 lncRNAs with the most up- or down-regulation were listed (Tables 5 and 6). Both the up-regulated and the down-regulated assembles had 6 lincRNAs (60%), 2 intronic lncRNAs (20%) and 2 anti-sense lncRNAs (20%). Accordingly, the proportions of intronic lncRNAs and anti-sense lncRNAs were higher in most changed top 10 lncRNAs (20%) than in all lncRNAs responsive to sulfur deprivation (9.7%).

### LncRNA target prediction, annotation and enrichment analysis

LncRNAs usually act on neighboring target genes, which is known as the *Cis* role of lncRNAs. We searched for coding genes 100 kb upstream and downstream of lncRNAs to predict putative *Cis* target genes of lncRNAs, followed by analyzing functions of these coding genes to annotate lncRNAs. KOBAS software was used to analyze the statistical enrichment of differentially expressed lncRNA target genes in KEGG pathways. Based on the results of significantly differentially expressed lncRNAs analysis, 99 pathways were found responsive to sulfur deprivation. The most enriched pathways including pentose phosphate pathway, plant hormone signal transduction, protein export, glutathione metabolism, amino acid metabolism and fatty acids metabolism. The analysis result was showed in a heat map indicating the expression levels of all pathways, and the entire pathways expression level heat map can be found in Supplementary Figure S2. The different pathways included pentose phosphate pathway, RNA polymerase and degradation, protein export, plant hormone signal transduction, base excision repair, and some metabolic pathways.

### LncRNAs related to photosynthesis

The differentially expressed photosynthetic proteins were investigated, and their regulating lncRNAs were located. In total, 23 photosynthesis-related mRNAs were found responsive to sulfur-deprived condition, and they were predicted to be the target genes of 36 sulfur deprivation-responsive lncRNAs. We classified lncRNA target genes into five types according to their positions in photosynthetic systems (Table 7). In detail, we were able to predict 14 lncRNAs related to Photosystem II, 10 lncRNAs related to Photosystem I, 7 lncRNAs related to photosynthetic electron transport, 3 lncRNAs related to ATP synthase and 2 lncRNAs related to cytochrome b6-f complex.

## Discussion

LncRNAs play important roles in various metabolic pathways in animal, plant, and yeast. Recent studies showed that they are closely related to cancer, nervous disease and autoimmune disease^8^,^9^,^10^,^11^,^12^. In plants, rice lncRNAs were reported to regulate the essential component photoperiod-sensitive male sterility of hybrid rice^17^, which has greatly contributed to the global increase of rice productivity and solution to food problem. The importance of lncRNA has been emphasized in many species, however, still remained completely unknown until this study in *C. Reinhardtii*. As a unicellular eukaryotic model organism, *C. reinhardtii* is an ideal model for studying chloroplast-based photosynthesis, structure, assembly and function of eukaryotic flagella (cilia) inherited from the common ancestor of plants and animals as a model of human cilia-related diseases. *C. reinhardtii* is also a model to study hydrogen production by green algae. In 2007, the complete genome of *C. reinhardtii* was sequenced, and microRNAs were also found in this species. In this study, high-throughput sequencing technique was used to scan the whole genome of *C. reinhardtii*, and 1,440 high confident long non-coding RNAs (936 lincRNAs, 310 intronic lncRNAs and 194 anti-sense lncRNAs) were identified. These lncRNAs have a median length of 509 nucleotides, and usually have 1~2 exons. In all, lncRNAs is shorter than mRNA, with fewer exons than mRNA. The transcription level of lncRNA is significantly lower than that of mRNAs, and the FPKM of most *C. reinhardtii* lncRNAs (85% of the 1,440) are less than 10. Compared with 98% lncRNAs spliced in human^30^, however, only 48.1% of *C. reinhardtii* lncRNAs were found spliced. This significant difference suggests the possible different origins and patterns of lncRNAs in *C. reinhardtii* and human.

Mercer *et al*. using tiling array successfully identified and characterized transcripts which were not detected and annotated by conventional sequencing approaches, because of their low or transient expression^38^. This suggested that our high throughput sequencing may also not include all lncRNAs in *C. reinhardtii*, and rare or transient lncRNAs and some lncRNAs responsive to special external stimuli were not identified under our experimental conditions. In this study, 111 lncRNAs can only be detected under sulfur-replete condition and 28 lncRNAs can only be detected under sulfur-deprived condition. Transcripts with long ORFs are considered more likely to encode proteins, and hence some filter principles exclude transcripts longer than 100 amino acids^18^ or 80 amino acids^39^ to remove transcripts with long ORFs, which are more likely to encode proteins. However, this restrictions may leave out some possible lncRNAs. That is why we did not discard the lncRNA candidates with long ORFs in the process of filtering.

The conservation of *C. reinhardtii* lncRNAs compared with other species was greatly affected by the integrality of genome assembly and the size of reference genomes. The *C. reinhardtii* have 64 lncRNAs conserved with *C. subellipsoidea*, and 169 lncRNAs conserved with *V. carteri* (Table 1). The longest *C. reinhardtii* conserved sequence length of lncRNA when compared with *C. subellipsoidea* and *V. carteri* is 433 and 485 nt, respectively. The number of conserved sequences possibly related to the genome size or/and sequencing and assembling completeness of the genome, whereas longer conserved sequences in *C. subellipsoidea* and *V. carteri* indicated that *C. reinhardtii* may have some lncRNAs that have higher conservation with *C. subellipsoidea* and *V. carteri* due to their close evolutionary relationship.

Even though some lncRNAs have verified functions, the molecular mechanism of how lncRNAs participate in bioprocesses is still largely unknown. For instance, lncRNAs can modulate protein-coding genes at transcription, post-transcription, and post-translation levels^8^,^10^,^13^,^19^,^20^. They can also affect the nearby genes positively or negatively by inducing chromatin remodeling or inhibiting RNA polymerase II recruitment^13^,^19^,^20^. What’s more, lncRNAs modulate alternative splicing by hybridization with targeted sense RNAs and block the recognition of the splice site of spliceosome^36^,^37^. In addition, some lncRNAs act as the precursor of miRNA, and can also interact with miRNA as a competing endogenous RNA^10^,^13^. Some lncRNAs are also able to bind with proteins to form RNA-protein complex to modulate protein activity or alter protein subcellular localization^40^,^41^. Therefore, by functions lncRNAs are classified as signaling, decoying, guiding and scaffolding lncRNAs^36^. At the same time, some lncRNAs have both *Cis* (acting on neighboring target genes) and *Trans* (identifying each other by the expression level) roles in regulating target genes. However, current prediction of *Trans* role of target gene needs more than 5 samples, so in this study only *Cis* targeted genes were considered. Further exploratory need more sequencing samples.

LncRNA is reported to modulate alternative splicing regulators in *Arabidopsis*^37^,^42^. Similar study on alternative splicing events was carried out to investigate *C. reinhardtii*. Surprisingly, the numbers and classification of mRNAs alternative splicing showed no difference. Future investigation of *C. reinhardtii* lncRNAs will continue with the integrative analysis of lncRNA, miRNA, mRNA and proteins, prediction and verification of lncRNA targets, and functions of differential expressed lncRNAs in the unicellular green alga *C. reinhardtii*.

The hydrogen production from green algae is a promising way to solve the global energy and environment problems. However, the main problem of hydrogen production from green algae is the inhibition of hydrogenase activity by oxygen, which results in the releasing of hydrogen from algal cells continuously for only a few seconds to a few minutes. Sulfur deprivation leads to sustained hydrogen production by *C. reinhardtii*, so the transcriptome and proteome of sulfur-deprived *C. reinhardtii* had been analyzed^24^,^25^,^26^,^31^. Transcriptome analysis revealed that sulfur deprivation resulted in repression of most transcripts encoding photosynthetic genes, except for *LHCBM9* (encoding a major light-harvesting polypeptide), and this indicated a remodeling of the photosystem II light-harvesting complex under sulfur deprivation^24^. Photosynthetic machinery was also one of the most changed components under sulfur deprivation in proteomic analysis, and other major changes consist of protein biosynthetic apparatus, molecular chaperones, and 20 S proteasomal components^26^. However, more regulatory mechanisms should be retrieved from other regulatory system, such as miRNAs, and lncRNAs. Thus lncRNAs responsive to sulfur deprivation investigated in this study.

Electrons which are necessary in green algae hydrogen production source from photosynthetic electron transport chain, and hydrogenase links with photosynthetic electron transport chain by ferredoxin (Fd). In addition, the oxygen produced by photosynthesis can inhibit enzyme activity of hydrogenase. Therefore, hydrogen production in green algae is closely related to photosynthesis. Our study discovered 36 lncRNAs targeting to 23 photosynthesis-related mRNAs responsive to sulfur deprivation. Obviously, this indicates that the lncRNAs may modulate the photosynthesis-related mRNAs. Sulfur deprivation repressed the expression of most photosynthesis-related mRNAs (22 of all the 23 photosynthesis-related mRNAs), except for *LHCSR3*. As to photosynthesis-related lncRNAs, 22 lncRNAs were up-regulated and 14 lncRNAs were down-regulated. LncRNAs regulated mRNAs in diverse ways. For instance, lncRNA XLOC_069036 was up-regulated after sulfur deprivation, but its target gene *PSBQ* was repressed; lncRNA XLOC_064073 was down-regulated, and its target gene *LHCBM1* was also down-regulated. Moreover, a considerable amount of mRNAs modulated by multiple lncRNAs, for example, the down-regulated mRNA *PSBW* was predicted to be regulated by tow up-regulated lncRNAs. On the other hand, lncRNAs always also had multiple targets, for example, the lncRNA XLOC_037244 was predicted to regulate *LHCBM2* as well as *LHCBM7*. These diverse expression patterns of lncRNAs and mRNAs indicated complicated regulation mechanism and various functions of *C. reinhardtii* lncRNAs. Thus, the 36 lncRNAs possibly regulate photobiological hydrogen production in *C. reinhardtii*. Our further research will focus on these sulfur deprivation-responsive and photosynthesis-related lncRNAs.

In summary, in this study we reported the first genome wide lncRNA profiling from a photosynthetic microorganism *Chlamydomonas reinhardtii*. Moreover, we identified 367 lncRNAs responsive to a promising simple hydrogen induction treatment, i.e., sulfur deprivation, including 36 photosynthesis-related lncRNAs with sulfur deprivation-responsive target genes. The lncRNA investigation may provide new insights into complicate regulations of biofuel production and thus extensive metabolic engineering could be conducted for potential improvements in the field of microalgal biofuels. Based on the predication of lncRNA and their targets, genetic manipulations focusing on these target genes will be employed for further potential improvements of hydrogen production in this model green microalga.

## Materials and Methods

### Growth and treatments of the algae

*C*. *reinhardtii* CC849 were obtained from *Chlamydomonas* Genetic Centre (Duke University, Durham, USA). The algae was grown in a Tris-Acetate-Phosphate (TAP) medium at 25 °C and under continuous cool-white fluorescent lamps (≈100 μmol photons·m^−2^·s^−1^). The sulfur deprivation treatment was performed according to Shu and Hu^26^. The algae were grown in liquid TAP until mid-log phase and algal cells were collected by centrifugation and washed twice with liquid TAP medium without sulfur (TAP-S, for 1 L of Medium: 2X Filner’s Beijernicks Solution 25 ml; 1 M Potassium Phosphate 1 ml; Trace mineral solution 1 ml; Tris-Base 2.42 g; adjust pH to 7.0 by Glacial Acetic Acid. Sulfur-deprivation media TAP-S were prepared by replacement of the S-salts by their chloride counterparts). Algal cells of equal numbers were resuspended in TAP or TAP-S under continuous illumination for 24 h, and then cell aliquots were collected for RNA isolation.

### Preparation of total RNA

Total RNA was extracted using RNAiso Plus reagent (Takara, Dalian, China) according to the manufacturer’s protocol. The algal cells cultured at 25 °C for 24 h in TAP and TAP-S were collected by centrifugation and treated with RNAiso Plus (Takara) immediately. Then rRNAs were removed by Ribo-zero™ rRNA Removal Kit (Epicentre, CA, USA) according to the manufacturer’s protocol. The quality of RNA was examined by using an Agilent 2100 Bioanalyzer.

### LncRNA library construction and high-throughput sequencing

Equivalent total RNAs from TAP and TAP-S cultured algal cells were used to construct the sulfur-replete and sulfur-deprived libraries by NEB Next^®^ Ultra™ Directional RNA Library Prep Kit for Illumina^®^ (NEB, USA) following manufacturer’s recommendations. Briefly, RNA was broken into fragments by divalent cations under elevated temperature in NEBNext First Strand Synthesis Reaction Buffer, and converted to first strand cDNA using random hexamer primer and M-MuLV Reverse Transcriptase. Second strand cDNA was synthesized subsequently using DNA Polymerase I and RNase H. dNTPs with dTTP were replaced by dUTP in the reaction buffer. Remaining overhangs were converted into blunt ends by exonuclease/polymerase. Adaptors with hairpin loop structure were ligated to prepare for hybridization after adenylation of DNA 3′ ends. For selecting 150~200 nucleotides cDNA fragments, the library were purified with AMPure XP system (Beckman Coulter, Beverly, USA)^12^. Then size-selected, adaptor-ligated cDNA was incubated with 3 μl USER Enzyme (NEB, USA) at 37 °C for 15 min followed by 5 min at 95 °C. Then PCR was performed to obtain enriched cDNA library. At last, products were purified (AMPure XP system) and assessed (Agilent Bioanalyzer 2100 system). The clustering of the index-coded samples was performed on a cBot Cluster Generation System using TruSeq PE Cluster Kit v3-cBot-HS (Illumia) according to the manufacturer’s instructions. After cluster generation, sequencing of the two libraries was performed on the Illumina HiSeq 2500 platform. Reads with more than 10% N (Unable to determine base information), with adapter sequence, or of low quality were removed from the raw reads to obtain clean reads. Finally, clean reads were compared with *C. reinhardtii* genome from NCBI using Tophat2^43^. The libraries preparation and deep sequencing were performed by Novogene Bioinformatics Technology Cooperation (Beijing, China).

### Bioinformatics analysis for identifying lncRNAs

The transcripts including mRNA, lncRNA and rRNA were assembled using Cufflinks^44^ and scripture^45^. The assembled transcripts detected in two or more samples or by two or more assemblers were selected for further analysis. Then transcripts less than 200 nucleotides were sorted out. The transcripts that have three or more reads coverage are chosen for further analyses. The sequences of remained transcripts were compared with the known non-coding RNAs (rRNA, tRNA, snRNA, snoRNA, pre-miRNA and pseudogenes) using Cuffcompare. The transcript sequences were also compared with the known mRNAs, and the candidate lincRNA, intronic lncRNA, anti-sense lncRNA were determined by class code obtained from Cuffcompare.

The transcripts were then aligned to NCBI protein database (NRDB) by CPC (Coding Potential Calculator)^46^ Transcripts with known protein domains were excluded by Pfam Scan^47^ according to Pfam HMM^48^. The intersection of transcripts filtered by CPC and Pfam Scan were considered as the lncRNAs.

### Quantitative Real-Time PCR (qRT-PCR) validation of lncRNAs

21 lncRNAs (FPKM range from 0 to 1423) were randomly chosen to validate the RNA-seq data. Total RNA were isolated respectively from algae cells cultivated in TAP and TAP-S for qRT-PCR using the RNAiso Plus reagent (Takara, Dalian, China) as previously described. First-strand cDNA was reverse transcribed by PrimeScript^TM^ RT reagent Kit with gDNA Eraser (Takara, Dalian, China). The qRT-PCR was performed using SYBR^®^ Premix Ex Taq^TM^ (Perfect Real Time) and ROX plus (Takara, Dalian, China). The U4 snRNA was used as the reference gene and all the primers used were as listed in Supplementary Table S2. The conditions for the PCR amplification were as follows: polymerase activation was conducted at 95 °C for 30 s; followed by 40 cycles at 95 °C for 5 s, 60 °C for 34 s. The specificity of the primer amplicons was tested by analysis of a melting curve and the PCR products were verified by gel purification and sequencing. This experiment was performed on QuantStudio^TM^ 6 Flex Real-Time PCR System (Life technologies) containing three technical replicates and three biological replicates.

### Distribution of lncRNAs along each chromosome

The *C. reinhardtii* lincRNAs, intronic lncRNAs and anti-sense lncRNAs were aligned to the genome of *C. reinhardtii* separately to obtain the lncRNA chromosome distribution. The lncRNAs were aligned by short blast, and the best hits were chosen to do subsequent analysis, with a summarized size of every 500 kb. The start sites of the lncRNA in the chromosome decided which zone this lncRNA was counted in. *C. reinhardtii* v5.5 genome from *Phytozome10.2* was used for analysis of chromosome distribution.

### LncRNA conservation in different species

The full length of all identified 1440 *C. reinhardtii* lncRNAs were used to blast against the genomes of *Arabidopsis thaliana*, *Homo sapiens*, *Coccomyxa subellipsoidea*, *Oryza sativa*, *Sorghum bicolor* and *Volvox carteri*, with the word-size = 5, and E value < 10E-5.

### KEGG enrichment analysis

KEGG is a bioinformatics database resource integrates genomic, chemical and systemic functional information to understand high-level functions and utilities of the biological system from molecular-level information, especially large-scale molecular datasets generated by high-throughput experimental technologies (http://www.genome.jp/kegg/). We used KOBAS software for testing the statistical enrichment of differential expressed lncRNA target genes in KEGG pathways^12^.

## Additional Information

**How to cite this article**: Li, H. *et al*. Genome-wide long non-coding RNA screening, identification and characterization in a model microorganism *Chlamydomonas reinhardtii*. *Sci. Rep*. **6**, 34109; doi: 10.1038/srep34109 (2016).

## Supplementary Material

Supplementary Information

Supplementary Data S2

Supplementary Data S3

Supplementary Data S4

## Figures and Tables

**Table 1:** Summary of the *C. reinhardtii* lncRNAs that are conserved in *Arabidopsis thaliana, Coccomyxa subellipsoidea, Oryza sativa, Sorghum bicolor* and *Volvox carteri*.

	Total Number	Length	Identity	coverage ≥20% Number	coverage ≥10% Number
*Arabidopsis*	38	28–356	85.71–100	11	21
*Coccomyxa*	64	25–433	83.33–100	12	27
*Oryza*	82	27–358	83.67–100	11	33
*Sorghum*	80	28–358	85.71–100	10	32
*Volvox*	169	25–485	78.54–100	33	74

**Table 2:** Classification of *C. reinhardtii* lncRNAs responsive to sulfur deprivation.

	Up-regulation	Down-regulation	Total
lincRNA	148	141	289 (78.7%)
intronic lncRNA	18	12	30 (8.2%)
anti-sense lncRNA	28	20	48 (13.1%)
Total	194	173	367 (100.0%)

**Table 3:** LncRNAs expressing only under sulfur-replete condition.

Transcript id	Gene_id	Length	CS_FPKM	C_FPKM	p value	q value	LncRNA type
TCONS_00047415	XLOC_015691	220	0	6.351	0.0002	0.0006	lincRNA
TCONS_00105300	XLOC_032923	259	0	2.234	0.0011	0.0033	lincRNA
TCONS_00132911	XLOC_041867	413	0	1.112	0.0001	0.0002	lincRNA
TCONS_00217931	XLOC_068457	278	0	2.468	0.0002	0.0006	lincRNA
TCONS_00228977	XLOC_071970	486	0	1.097	0.0001	0.0002	lincRNA
TCONS_00232151	XLOC_073149	235	0	3.719	0.0019	0.0054	lincRNA

**Table 4:** LncRNAs expressing only under sulfur-deprived condition.

Transcript id	Gene_id	Length	CS_FPKM	C_FPKM	p value	q value	LncRNA type
TCONS_00057160	XLOC_018017	328	2.220	0	0.0001	0.0002	lincRNA
TCONS_00126205	XLOC_038805	218	4.456	0	0.0055	0.0141	lincRNA
TCONS_00138339	XLOC_043052	244	3.658	0	0.0008	0.0024	lincRNA
TCONS_00141761	XLOC_043722	272	3.555	0	0.0001	0.0002	lincRNA
TCONS_00175099	XLOC_054528	736	26.714	0	0.0001	0.0002	lincRNA
TCONS_00198720	XLOC_061964	234	2.987	0	0.0055	0.0141	lincRNA
TCONS_00233133	XLOC_073215	246	3.501	0	0.0010	0.0030	intronic_lncRNA
TCONS_00246373	XLOC_077711	203	5.842	0	0.0120	0.0277	lincRNA
TCONS_00249259	XLOC_079237	276	2.875	0	0.0001	0.0004	lincRNA
TCONS_00249661	XLOC_079440	823	3.583	0	0.0001	0.0002	lincRNA

**Table 5:** Part of up-regulated lncRNAs under sulfur-deprived condition.

Transcript_id	Gene id	Length	CS_FPKM	C_FPKM	log2 (foldchange)	p value	q value	LncRNA type
TCONS_00193953	XLOC_060741	738	344.424	0.864	8.63935	0.0001	0.0002	lincRNA
TCONS_00165611	XLOC_051494	909	185.423	0.507	8.5152	0.0001	0.0002	lincRNA
TCONS_00015255	XLOC_002485	1620	49.302	0.199	7.96E + 00	0.0001	0.0002	lincRNA
TCONS_00193429	XLOC_060041	1442	23.070	0.102	7.82231	0.0038	0.0101	lincRNA
TCONS_00247239	XLOC_078200	865	135.347	0.662	7.68E + 00	0.0001	0.0002	lincRNA
TCONS_00217604	XLOC_068375	771	32.579	0.538	5.92135	0.0001	0.0002	lincRNA
TCONS_00128008	XLOC_038901	2286	7.685	0.147	5.704	0.0004	0.0012	anti-sense_lncRNA
TCONS_00108002	XLOC_033894	404	43.829	0.904	5.5991	0.0089	0.0215	intronic_lncRNA
TCONS_00083547	XLOC_026230	815	6.374	0.293	4.44E + 00	0.0084	0.0203	intronic_lncRNA
TCONS_00121133	XLOC_037215	2393	4.203	0.198	4.40974	0.0001	0.0002	anti-sense_lncRNA

**Table 6:** Part of down-regulated lncRNAs under sulfur-deprived condition.

Transcript_id	Gene id	Length	CS_FPKM	C_FPKM	log2 (foldchange)	p value	q value	LncRNA type
TCONS_00250327	XLOC_079799	735	2.064	166.072	−6.33024	0.0001	0.0002	lincRNA
TCONS_00239315	XLOC_075287	1279	0.125	8.300	−6.06E + 00	0.0061	0.0153	anti-sense_lncRNA
TCONS_00207522	XLOC_065013	577	20.183	695.828	−5.10751	0.0001	0.0002	lincRNA
TCONS_00101007	XLOC_031585	1923	3.389	86.689	−4.67679	0.0001	0.0002	anti-sense_lncRNA
TCONS_00124655	XLOC_039515	1346	17.007	353.366	−4.37695	0.0001	0.0002	intronic_lncRNA
TCONS_00236134	XLOC_074211	721	1.162	23.524	−4.34E + 00	0.0001	0.0002	lincRNA
TCONS_00224847	XLOC_070799	293	5.670	77.785	−3.77819	0.0001	0.0002	intronic_lncRNA
TCONS_00054981	XLOC_018225	868	0.275	3.752	−3.77E + 00	0.0005	0.0015	lincRNA
TCONS_00005316	XLOC_004385	321	3.244	40.337	−3.63628	0.0001	0.0002	lincRNA
TCONS_00211477	XLOC_066226	512	10.660	129.862	−3.60671	0.0001	0.0002	lincRNA

**Table 7:** Sulfur deprivation-responsive lncRNAs targeting photosynthetic electron transport chain proteins.

Location	Gene	Description	log2 (fold change)	p value	LncRNA ID	CS FPKM	C FPKM	log2 (fold change)	p value
Photosystem II	PSBQ↓	oxygen evolving enhancer protein 3	−3.4316	5.00E-05	XLOC_069036↑	3.17	1.62	0.9650	0.00095
	PSBW↓	photosystem II reaction center W protein	−2.7594	5.00E-05	XLOC_070848↑	14.60	7.19	1.0226	0.0004
					XLOC_070910↑	1.64	0.28	2.5673	0.00255
	CPLD45↓	hypothetical protein	−3.9667	5.00E-05	XLOC_015691↓	0.00	6.35	nd	0.00015
	LHCBM5↓	chlorophyll a-b binding protein of LHCII	−4.0723	5.00E-05	XLOC_052680↓	3.26	30.00	−3.2036	5.00E-05
	LHCBM2↓	light-harvesting protein of photosystem II	−2.5739	5.00E-05	XLOC_037917↓	2.29	8.18	−1.8354	5.00E-05
					XLOC_037244↑	1.26	0.42	1.5935	0.00085
					XLOC_037246↓	1.37	7.67	−2.4791	0.00345
					XLOC_037215↑	4.20	0.20	4.4097	5.00E-05
	LHCBM7↓	chlorophyll a-b binding protein of LHCII	−4.1974	5.00E-05	XLOC_037244↑	1.26	0.42	1.5935	0.00085
					XLOC_037246↓	1.37	7.67	−2.4791	0.00345
					XLOC_037917↓	2.29	8.18	−1.8354	5.00E-05
	LHCBM1↓	chlorophyll a-b binding protein of LHCII	−3.1900	5.00E-05	XLOC_064073↓	0.73	6.04	−3.0516	0.0001
	LHCB4↓	chlorophyll a-b binding protein of photosystem II	−3.6009	5.00E-05	XLOC_050637↓	11.34	15.85	−0.4837	0.0146
Photosystem I	PSAE↓	photosystem I 8.1 kDa reaction center subunit IV	−3.7333	5.00E-05	XLOC_019240↑	2.02	1.11	0.8697	0.00985
	PSAG↓	photosystem I reaction center subunit V	−3.3373	5.00E-05	XLOC_074957↓	3.16	7.02	−1.1509	5.00E-05
	PSAI↓	photosystem I reaction center subunit VIII	−2.5505	5.00E-05	XLOC_021615↑	2.65	0.34	2.9641	5.00E-05
					XLOC_021656↓	6.55	24.07	−1.8769	5.00E-05
	PSAL↓	photosystem I reaction center subunit XI	−2.7222	5.00E-05	XLOC_024559↑	81.57	47.16	0.7905	0.00895
	LHCSR3↑	stress-related chlorophyll a/b binding protein 3	8.6919	5.00E-05	XLOC_048026↓	2.91	5.87	−1.0121	0.00055
	LHCA3↓	light-harvesting chlorophyll-a/b protein of photosystem I, type III	−3.2610	5.00E-05	XLOC_069667↑	1.91	0.75	1.3533	5.00E-05
	LHCP2↓	regulatory chlorophyll a/b binding protein	−0.6977	0.00065	XLOC_010876↑	36.05	13.87	1.3782	5.00E-05
					XLOC_011835↑	4.11	1.07	1.9458	0.00025
	LHCA9↓	light-harvesting protein of photosystem I	−2.7130	5.00E-05	XLOC_032037↑	17165.10	5873.25	1.5473	5.00E-05
Photosynthetic electron transport	PETF↓	apoferredoxin	−4.4579	5.00E-05	XLOC_033091↑	97.04	7.05	3.7820	5.00E-05
					XLOC_033128↑	9.32	4.99	0.9032	0.00045
					XLOC_033894↑	43.83	0.90	5.5991	0.0089
	FDX5↓	apoferredoxin	−8.1388	5.00E-05	XLOC_027503↑	4.39	1.32	1.7350	0.00315
	FNR1↓	ferredoxin-nadp reductase	−4.4283	5.00E-05	XLOC_051494↑	185.42	0.51	8.5152	5.00E-05
					XLOC_051478↑	12.03	7.14	0.7535	0.0002
	CYC4↓	chloroplast cytochrome c	−1.1520	0.0018	XLOC_029266↓	2.59	5.40	−1.0604	0.0022
ATP synthase	ATPD↓	chloroplast ATP synthase delta chain	−2.2429	5.00E-05	XLOC_069667↑	1.91	0.75	1.3533	5.00E-05
	ATPG↓	CF0 ATP synthase subunit II precursor	−1.8908	5.00E-05	XLOC_052299↓	54.05	136.09	−1.3323	0.00025
					XLOC_051836↑	5.42	3.53	0.6175	0.0108
cytochrome b6-f complex	PETC↓	rieske iron-sulfur subunit of the cytochrome b6f complex, chloroplast precursor	−2.4417	0.00005	XLOC_057939↓	4.26	14.78	−1.7937	5.00E-05
					XLOC_058281↑	3.04	0.45	2.7454	0.00005
