# Supplementary material for: Genome-wide long non-coding RNA screening, identification and characterization in a model microorganism Chlamydomonas reinhardtii
Source: Sci Rep. 2016 Sep 23;6:34109. doi: 10.1038/srep34109 (PMC5034253; doi:10.1038/srep34109)
Supplement: Supplementary Information [file srep34109-s1.pdf]

Genome-wide long non-coding RNA screening, identification and characterization in a model microorganism *Chlamydomonas reinhardtii*

Hui Li[1,2#], Yuting Wang [2,3#], Meirong Chen[1,2#], Peng Xiao[1,2], Changxing Hu[1,2], Zhiyong Zeng[1,2], Chaogang Wang[1,2], Jiangxin Wang[1,2], Zhangli Hu[1,2]*

[1]Guangdong Technology Research Center for Marine Algal Bioengineering, Guangdong Key Laboratory of Plant Epigenetic, College of Life Sciences, Shenzhen University, Shenzhen 518060, P.R. China

[2]Shenzhen Key Laboratory of Marine Bioresource & Eco-environmental Science, College of Life Sciences, Shenzhen University, Shenzhen 518060, P.R. China

[3]Key Laboratory of Optoelectronic Devices and Systems of Ministry of Education and Guangdong Province, College of Optoelectronic Engineering, Shenzhen University, Shenzhen 518060, P.R. China

* Corresponding author at: College of Life Science, Shenzhen University, Shenzhen 518060, PR China.

Tel.: +86 755 26557244; Fax: +86 755 26534274.

E-mail address: huzl@szu.edu.cn (Z. Hu).

# These authors contributed equally to this work.

Supplementary Information

Supplementary Data S1. Classification of mapped reads into the known categories of RNAs.

Supplementary Data S2. The sequences of all 1,440 lncRNAs identified by CPC and Pfam.

Supplementary Data S3. Conservation blast results of all 1,440 lncRNAs.

Supplementary Data S4. Significantly differentially expressed lncRNAs under sulfur-replete and sulfur-deprived conditions.

Supplementary Figure S1. Statistics of alternative splicing events. (1) TSS: Alternative 5' first exon. (2) TTS: Alternative 3' last exon. (3) SKIP: Skipped exon. (4) XSKIP: Approximate SKIP. (5) MSKIP: Multi-exon SKIP. (6) XMSKIP: Approximate MSKIP. (7) IR: Intron retention. (8) XIR: Approximate IR. (9) MIR: Multi-IR. (10) XMIR: Approximate MIR. (11) AE: Alternative exon ends (5', 3', or both). (12) XAE: Approximate AE. X axis: numbers of AS events. Y axis: events types.

Supplementary Figure S2. KEGG enrichment analysis of lncRNA target genes significantly differentially expressed under sulfur-replete and sulfur-deprived conditions. Colors represents the expression levels of metabolic pathways. Red represents a high expressing metabolic pathway. Green represents a relatively low expressing metabolic pathway. The left column (+S) is the sulfur-replete sample and the right column (-S) is the sulfur-deprived sample. The numbers of significant different genes contained in each metabolic pathway are indicated in the parentheses

after the label of each metabolic pathway.

Supplementary Table S1. Reads mapping to the reference genome.

Supplementary Table S2. The qRT-PCR primers used in this study.

Data S1. Classification of mapped reads into the known categories of RNAs.

Sample name	C1	C2	CS1	CS2
**mRNA**	27138309 (75.53%)	30405244 (76.45%)	23268563 (66.86%)	22508944 (68.36%)
**pseudogene**	2680 (0.01%)	2840 (0.01%)	11287 (0.03%)	11387 (0.03%)
**rRNA**	884723 (2.46%)	813147 (2.04%)	34256 (0.10%)	31131 (0.09%)
**tRNA**	4462 (0.01%)	4898 (0.01%)	4630 (0.01%)	3363 (0.01%)
**Others**	7897957 (21.98%)	8543944 (21.48%)	11485580 (33.00%)	10373498 (31.50%)

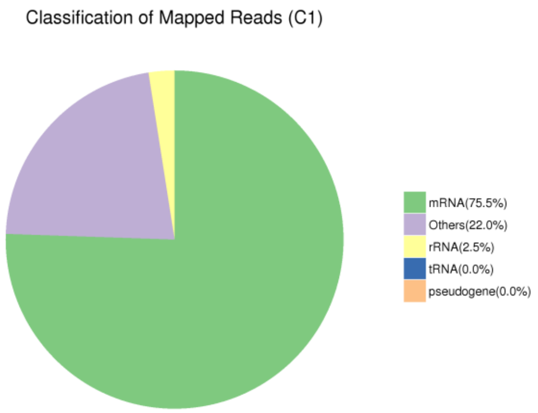

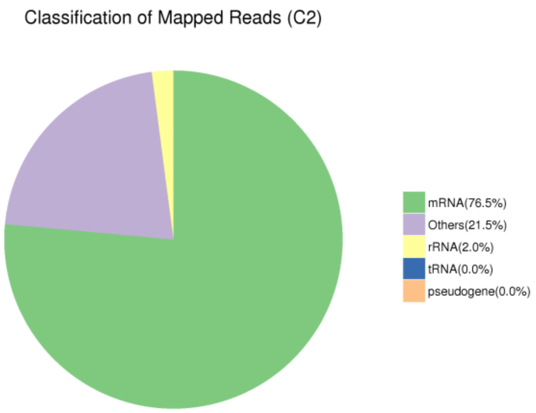

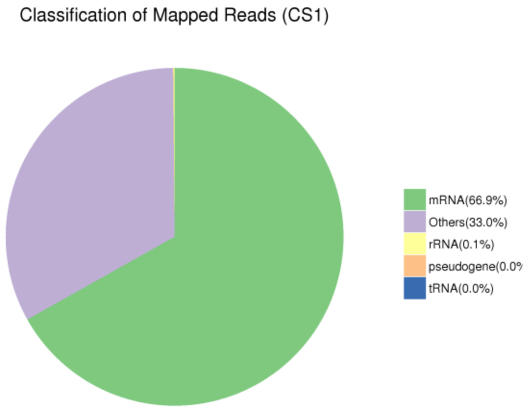

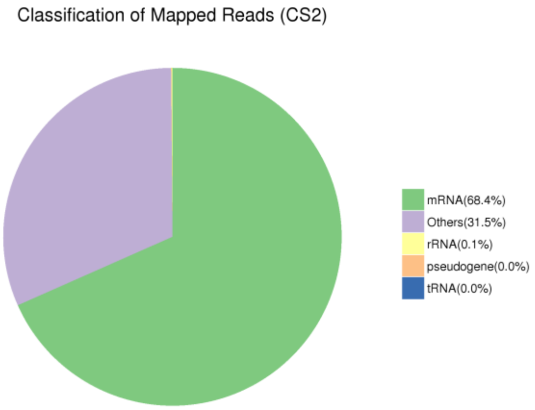

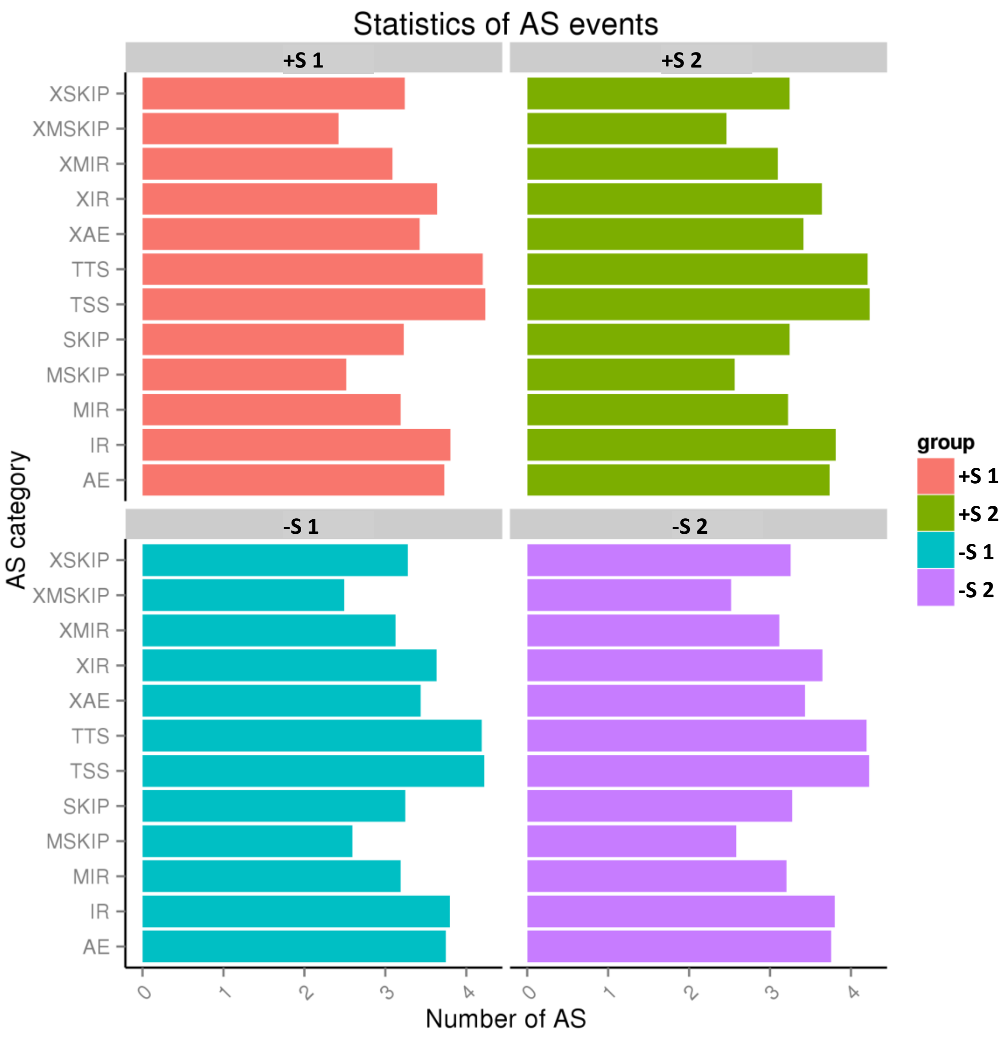

Figure S1. Statistics of alternative splicing events.
(1) TSS: Alternative 5' first exon. (2) TTS: Alternative 3' last exon. (3) SKIP: Skipped exon. (4) XSKIP: Approximate SKIP. (5) MSKIP: Multi-exon SKIP. (6) XMSKIP: Approximate MSKIP. (7) IR: Intron retention. (8) XIR: Approximate IR. (9) MIR: Multi-IR. (10) XMIR: Approximate MIR. (11) AE: Alternative exon ends (5', 3', or both). (12) XAE: Approximate AE. X axis: numbers of AS events. Y axis: events types.

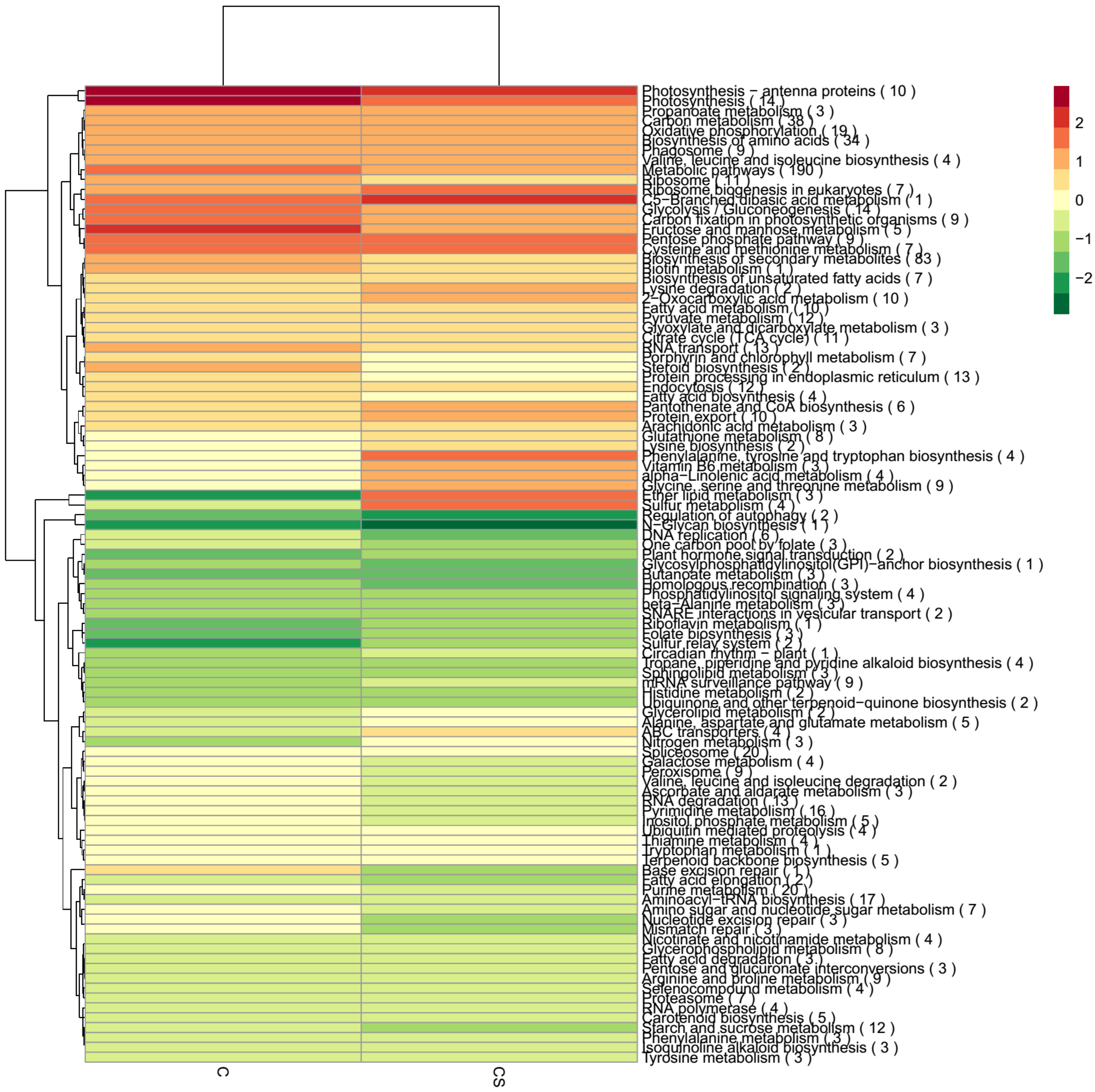

Photosynthesis − antenna proteins (10)
Photosynthesis (14)
Propanoate metabolism (3)
Carbon metabolism (38)
Oxidative phosphorylation (19)
Biosynthesis of amino acids (34)
Phagosome (9)
Valine, leucine and isoleucine biosynthesis (4)
Metabolic pathways (190)
Ribosome (11)
Ribosome biogenesis in eukaryotes (7)
C5−Branched dibasic acid metabolism (1)
Glycolysis / Gluconeogenesis (14)
Carbon fixation in photosynthetic organisms (9)
Fructose and mannose metabolism (5)
Pentose phosphate pathway (9)
Cysteine and methionine metabolism (7)
Biosynthesis of secondary metabolites (83)
Biotin metabolism (1)
Biosynthesis of unsaturated fatty acids (7)
Lysine degradation (2)
2−Oxocarboxylic acid metabolism (10)
Fatty acid metabolism (10)
Pyruvate metabolism (12)
Glyoxylate and dicarboxylate metabolism (3)
Citrate cycle (TCA cycle) (11)
RNA transport (13)
Porphyrin and chlorophyll metabolism (7)
Steroid biosynthesis (2)
Protein processing in endoplasmic reticulum (13)
Endocytosis (12)
Fatty acid biosynthesis (4)
Pantothenate and CoA biosynthesis (6)
Protein export (10)
Arachidonic acid metabolism (3)
Glutathione metabolism (8)
Lysine biosynthesis (2)
Phenylalanine, tyrosine and tryptophan biosynthesis (4)
Vitamin B6 metabolism (3)
alpha−Linolenic acid metabolism (4)
Glycine, serine and threonine metabolism (9)
Ether lipid metabolism (3)
Sulfur metabolism (4)
Regulation of autophagy (2)
N−Glycan biosynthesis (1)
DNA replication (6)
One carbon pool by folate (3)
Plant hormone signal transduction (2)
Glycosylphosphatidylinositol(GPI)−anchor biosynthesis (1)
Butanoate metabolism (3)
Homologous recombination (3)
Phosphatidylinositol signaling system (4)
beta−Alanine metabolism (3)
SNARE interactions in vesicular transport (2)
Riboflavin metabolism (1)
Folate biosynthesis (3)
Sulfur relay system (2)
Circadian rhythm − plant (1)
Tropane, piperidine and pyridine alkaloid biosynthesis (4)
Sphingolipid metabolism (3)
mRNA surveillance pathway (9)
Histidine metabolism (2)
Ubiquinone and other terpenoid−quinone biosynthesis (2)
Glycerolipid metabolism (2)
Alanine, aspartate and glutamate metabolism (5)
ABC transporters (4)
Nitrogen metabolism (3)
Spliceosome (20)
Galactose metabolism (4)
Peroxisome (9)
Valine, leucine and isoleucine degradation (2)
Ascorbate and aldarate metabolism (3)
RNA degradation (13)
Pyrimidine metabolism (16)
Inositol phosphate metabolism (5)
Ubiquitin mediated proteolysis (4)
Thiamine metabolism (4)
Tryptophan metabolism (1)
Terpenoid backbone biosynthesis (5)
Base excision repair (1)
Fatty acid elongation (2)
Purine metabolism (20)
Aminoacyl−tRNA biosynthesis (17)
Amino sugar and nucleotide sugar metabolism (7)
Nucleotide excision repair (3)
Mismatch repair (3)
Nicotinate and nicotinamide metabolism (4)
Glycerophospholipid metabolism (8)
Fatty acid degradation (3)
Pentose and glucuronate interconversions (3)
Arginine and proline metabolism (9)
Selenocompound metabolism (4)
Proteasome (7)
RNA polymerase (4)
Carotenoid biosynthesis (5)
Starch and sucrose metabolism (12)
Phenylalanine metabolism (3)
Isoquinoline alkaloid biosynthesis (3)
Tyrosine metabolism (3)
C
CS
2
1
0
−1
−2

Supplementary Figure S2. KEGG enrichment analysis of lncRNA target genes significantly differentially expressed under sulfur-replete and sulfur-deprived conditions. Colors represents the expression levels of metabolic pathways. Red represents a high expressing metabolic pathway. Green represents a relatively low expressing metabolic pathway. The left column (+S) is the sulfur-replete sample and the right column (-S) is the sulfur-deprived sample. The numbers of significant different genes contained in each metabolic pathway are indicated in the parentheses after the label of each metabolic pathway.

Table S1. Reads mapping to the reference genome.

Sample name	C1	C2	CS1	CS2
Total reads	91425938	102815474	90389134	84422494
Total mapped	86442571 (94.55%)	97457645 (94.79%)	84237229 (93.19%)	79078218 (93.67%)
Multiple mapped	15371828 (16.81%)	18795812 (18.28%)	15276981 (16.9%)	13470770 (15.96%)
Uniquely mapped	71070743 (77.74%)	78661833 (76.51%)	68960248 (76.29%)	65607448 (77.71%)
Read-1	35792638 (39.15%)	39624245 (38.54%)	34881093 (38.59%)	33097592 (39.2%)
Read-2	35278105 (38.59%)	39037588 (37.97%)	34079155 (37.7%)	32509856 (38.51%)
Reads map to '+'	35574776 (38.91%)	39386214 (38.31%)	34517033 (38.19%)	32828695 (38.89%)
Reads map to '-'	35495967 (38.82%)	39275619 (38.2%)	34443215 (38.11%)	32778753 (38.83%)
Non-splice reads	57869187 (63.3%)	64766137 (62.99%)	53072328 (58.72%)	50416096 (59.72%)
Splice reads	13201556 (14.44%)	13895696 (13.52%)	15887920 (17.58%)	15191352 (17.99%)

Supplementary Table S2. The qRT-PCR primers used in this study.

Name	Primer Sequence
U4-Forward Primer	CAAAAGGCCCGACAGAAAT
U4-Reverse Primer	GTGAGGTCTAACCGAGTCGC
XLOC_065013-Forward Primer	CCGTCGTGAGACAGGTTAGT
XLOC_065013-Reverse Primer	CTTAGAGGCGTTCAGTCATTAG
XLOC_032845-Forward Primer	CGCCTATGCCCTATGAT
XLOC_032845-Reverse Primer	GTGTTTGTCTCGCACTCG
XLOC_065816-Forward Primer	CGTATTCAAATCACGCACAG
XLOC_065816-Reverse Primer	GTCGGGCAACATCTCAACT
XLOC_039822-Forward Primer	TAAAGGTCCTGGGTTCG
XLOC_039822-Reverse Primer	CCGCCTCATCCATTCAC
XLOC_024082-Forward Primer	TTGCGTCGCTGGCTGAAGA
XLOC_024082-Reverse Primer	AAAGTTAGCACGGGTGAAGTAG
XLOC_071550-Forward Primer	CAACGAGGAAACTCTACGC
XLOC_071550-Reverse Primer	CCTGTGGGACCCTGACT
XLOC_072235-Forward Primer	CGCCCTCAGCCCTTCAA
XLOC_072235-Reverse Primer	GGGACGCAGCAAACAGG
XLOC_063143-Forward Primer	TACACCCTCTTCCACAACCG
XLOC_063143-Reverse Primer	CCCTACCTCCCGCAGATT
XLOC_053622-Forward Primer	CCCTTGTCACATTCGTGTTTAGC
XLOC_053622-Reverse Primer	GGTATCAAGTAATCCGACTGGTG
XLOC_039515-Forward Primer	TGTCTGAGGTTCGCCATTA
XLOC_039515-Reverse Primer	CAGTCTGCGTGCGTGTA
XLOC_070799-Forward Primer	ACCAAACTTCGCTGATGC
XLOC_070799-Reverse Primer	GTCGCTTGTGAGACGTGAT
XLOC_041576-Forward Primer	TCAAACGCAAGCCACGAC
XLOC_041576-Reverse Primer	ACGCCTCATCCTCTTTCTAC
XLOC_077711-Forward Primer	GGTCCTTAGAGGGAGGCATT
XLOC_077711-Reverse Primer	CCATTCTGACACCTAAGCACA
XLOC_011459-Forward Primer	AGCATACCAACCCATCACA
XLOC_011459-Reverse Primer	CCTCCCTAAGGCAACCAC
XLOC_066806-Forward Primer	CCTGCCTCCTGCGTCTA
XLOC_066806-Reverse Primer	CCTCCTGGGTCCATTCC
XLOC_041867-Forward Primer	GCTCTGCTTCGCCCTTCA
XLOC_041867-Reverse Primer	TGCCGCACCCTTCTTCG
XLOC_014186-Forward Primer	CCCCAAACTAAACAGAACGA
XLOC_014186-Reverse Primer	ACCGCATACAGAAACAAAGC
XLOC_067316-Forward Primer	GGACTGAGTGAAGGTGGATG
XLOC_067316-Reverse Primer	ATACCTACGCACGGACCAA
XLOC_079845-Forward Primer	CACAGCAACAGCGCCGTGAG
XLOC_079845-Reverse Primer	GCAGTAGCCCGACCAGGATG
XLOC_071546-Forward Primer	CCCACGAACGATAGGTAA
XLOC_071546-Reverse Primer	CTAGGGAAACGCCACATT
XLOC_078800-Forward Primer	CCCGCTGCAACCTAGAAAG
XLOC_078800-Reverse Primer	CGAAACTACAACCGCCAAG
